# Chemical Modification and Performance Evaluation of an Agro‐Waste for Column Mode Removal of Textile Dyes

**DOI:** 10.1002/gch2.201700131

**Published:** 2018-07-23

**Authors:** Gita Samchetshabam, Satya Prakash Shukla, Jyoti Matolia, Chandra Prakash, Vidyashree Bharti, Arvind Rajdeo Singh

**Affiliations:** ^1^ Aquatic Environment and Health Management Division ICAR–Central Institute of Fisheries Education Mumbai 400061 Maharashtra India; ^2^ Department of Physics G. N. Khalsa College Matunga (East) ,400019 Mumbai India

**Keywords:** column bed, kinetic models, sugarcane bagasse, textile dyes

## Abstract

The performance of a newly designed column bed device packed with chemically modified agro‐waste (sugarcane bagasse) is evaluated for efficient removal of two textile dyes, Optilan yellow and Lanasyn brown and textile industry dye effluent. The parameters used for performance evaluation are removal efficiency (*R*%), adsorption capacity (*q*), and breakthrough (*C*_e_/*C*_0_). The column exhibits >90% removal of both the dyes and >80% removal of textile industry dye effluent. The experimental data are fitted to three models, viz., Thomas, Adams–Bohart, and Yoon–Nelson for understanding the kinetic behavior of adsorption. The results reveal that the Yoon–Nelson model is the best‐fitted model for both the dyes with *R* ^2^ values 0.995 and 0.905 for Optilan yellow and Lanasyn brown, respectively. However, Thomas and Yoon–Nelson fit the best for textile industry effluent. Overall, this investigation provides baseline information about the column mode removal of textile dyes using the agro‐waste material as an adsorbent. The Fourier transform infrared spectroscopy spectra show the characteristic vibrational mode of C—H stretching of hydroxyl group around 2800–3000 cm^−1^. The change in the vibrational pattern is evident after alginic acid treatment, indicating delignification of the bagasse after treatment.

## 1 Introduction

Textile dyes are water‐soluble synthetic compounds used in textile industry for the coloration of fibers. The coloration process requires a large volume of water which contains unadsorbed dye molecules of various synthetic dyes. The untreated dye‐house wastewater poses a threat to the terrestrial and aquatic ecosystem and therefore, requires a pretreatment process for dye‐containing wastewater before its discharge to the surrounding. The synthetic dyes consist of azo, methane, nitro, and carbonyl groups which make them comparatively more recalcitrant to biological degradation processes.^1^, ^2^, ^3^, ^4^ It is estimated that globally 280 000 tons of textile dyes are discharged in the dye‐containing industrial effluents every year.^5^ Hence, significant amounts of dyes come in the effluent stream and pollute the waters. In general, dyes have low toxicity in mammals and aquatic organisms,^6^ but products formed by their biodegradation, mainly aromatic amines from the anaerobic reduction of dyes, can be harmful.^7^, ^8^ These discharges of synthetic dye into the aquatic system have generated much concern due to its reported genotoxic, mutagenic, teratogenic, and carcinogenic effects.^9^, ^10^ The release of dye bearing wastewater into natural rivers and streams poses severe problems to the aquatic organisms by disturbing the normal processes of nutrient cycling due to toxic effects on different trophic levels. Given an alarming increase in consumption and subsequent discharge of textile dyes by industries, an emphasis is laid upon the development of low‐cost technologies for the removal of textile dyes from wastewater.^11^ Removal of synthetic dyes from effluents is essential, not only to protect human health but also for the protection of water resources. However, dye‐containing wastewater is a tedious process to treat as the dyes are recalcitrant organic molecules, they are resistant to aerobic digestion, and are sTable to light, heat, and oxidizing agents.^11^ There are numerous treatment methods and techniques available in the literature for the removal of dye molecules from water. Most common methods include electrochemical, ion exchange, coagulation–flocculation, ozonation, adsorption, microbial bioremediation, etc.^12^, ^13^, ^14^ Among the different methods for dye molecule removal from effluents, biosorption – the adsorption of pollutants by nonliving biomass, has been strongly recommended by researchers worldwide as an efficient and economically sustainable technology for the removal of synthetic dyes from industrial effluents.^15^ Several numbers of inexpensive and abundant biosorbents, especially agro‐waste materials, as well as municipal and industrial wastes, have been utilized by researchers for the removal of synthetic dyes^9^, ^10^, ^11^, ^16^, ^17^ from the aqueous solutions.

Utilization of agricultural wastes for wastewater treatment is a novel and environment‐friendly approach which could be helpful in providing a solution for the problem of solid waste disposal. Several researchers used sugarcane bagasse as an adsorbent to remove different dyes from wastewater, especially in the batch study.^3^, ^18^, ^19^, ^20^, ^21^ A higher quantity of discarded sugarcane bagasse and adsorptive nature of sugarcane bagasse have increased its desirability for the remedy of environmental pollution in different ways.^18^ In India, an appreciable volume of bagasse is available as a waste material which can be used as an efficient adsorbent material for removing dyes. Sugarcane bagasse has cellulose (46.0%), hemicellulose (24.5%), lignin (19.95%), fat and waxes (3.5%), ash (2.4%), silica (2.0%), and others (1.7%) as main components.^22^ Polysaccharides of sugarcane bagasse are biopolymers with many hydroxyl and phenolic groups. These polysaccharides can be chemically altered to form new compounds with various properties.^18^, ^23^ During recent past, few studies were accomplished and reported on the removal of textile dyes as compared to batch mode removal. In an earlier attempt, Mohammad et al.^24^ assessed the performance of a prototype for removal of methylene blue using agro‐wastes such as sugarcane bagasse, sand, black tea beans,^24^ however, this investigation was conducted at a comparatively smaller scale using flow rate of 1.0 mL min^−1^, column height of 7.5 cm, and adsorbent weight of 1.4 gm. Therefore, the novelty of the present investigation lies in the potential of the prototype developed for upscaling up to the desired extent. The overall cost of upscaling will be negligible as the agro‐waste (sugarcane bagasse) used is available mostly free of cost. Another study on fixed column bed using magnetic clay as the adsorbent to remove golden XGL dye with different flow rates of 1.8, 2.7, 3.6 mL min^−1^ and at dye concentrations of 50, 75, 100 ppm, was performed by Nausheen et al..^25^ A review of the literature for column mode removal of textile dyes using chemically modified agro‐waste shows that the number of reports on column mode remediation of textile dyes is comparatively smaller than the batch mode studies. Further, most of the column mode studies were performed on smaller prototypes where the upscaling was either not feasible or was not cost‐effective. In above context, the novelty of the column bed developed during the present investigation lies in its characteristics such as the use of a chemically modified adsorbent, performance evaluation using real textile effluent, higher column bed height (45.72 cm) and diameter (16.5 cm), and a higher average flow rate of 250 mL min^−1^.

Based on the above background, the present study was carried out on the utilization of chemically modified sugarcane bagasse in the column‐based filtration unit for removal of Optilan yellow (acid dye) and Lanasyn brown textile dye (metal complex forming dye) from aqueous solutions. The data of the investigation will provide a better understanding of the efficiency of sugarcane bagasse as an adsorbent for the treatment of dye‐house wastewater. The information and data will help in further upgradation of technologies on dye‐house wastewater treatment.

## 2 Results and Discussion

### 2.1 Removal Efficiency of Novel Column‐Based Filtration Unit

**Figure 1:**
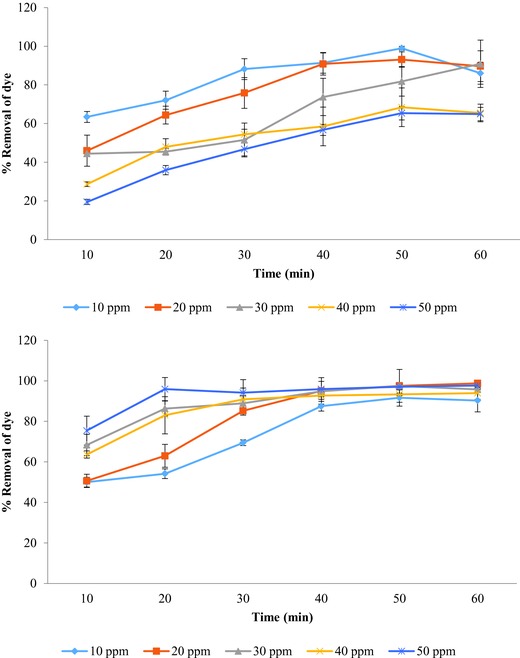
Effect of initial dye concentration on percentage removal (±SE) of Optilan yellow (above) and Lanasyn brown (below) by chemically modified sugarcane bagasse in column‐based filtration unit (temperature 30 ± 2 °C).

Removal of hazardous soluble chemicals from wastewater using low‐cost bioadsorbents has opened a new paradigm in the field of wastewater treatment technology.^26^ The present investigation aimed to develop a low‐cost treatment process for dye removal from water using agro‐waste sugarcane bagasse for the removal of two recalcitrant textile dyes: Optilan yellow and Lanasyn brown. The reason for selection of column mode for the present study was the insufficient availability of information about removal technologies based upon column bed reactors consisting of sugarcane bagasse as adsorbents. The bagasse was pretreated with a low concentration of the biopolymer, i.e., alginic acid, to enhance the adsorption capacity. Alginic acid was used as adsorbent since it is nontoxic, selective, efficient, and inexpensive, and consists of abundant functional groups with affinity for dye. The present study on utilization of agro‐waste like sugarcane bagasse for the removal of Optilan yellow and Lanasyn brown showed a significant reduction (maximum 98%) in dye concentration in all the cases in a column‐based system (**Figure** **1** and **Tables** **1** and **2**). However, in an earlier investigation, Sharma and Nandi^3^ reported 95% removal of Malachite green dye in a batch system using sugarcane bagasse particles as an adsorbent. Sadaf and Bhatti^21^ studied the potential application of sugarcane bagasse as cost‐effective biomass for the treatment of real textile effluents and observed maximum chemical oxygen demand (COD) removal of 72.4–80.8% from dye effluents. As compared to the above reports, chemically treated sugarcane bagasse showed an appreciable removal of both the dyes.

**Table 1:** Percentage removal of Optilan yellow dye solution in different times by chemically modified sugarcane bagasse in column‐based filtration unit (temperature 30 ± 2 °C)

Time [min]	Concentration [ppm]				
	10	20	30	40	50
10	63.44 ± 2.84^b,c)^	45.98 ± 8.05^d,e)^	44.45 ± 1.01a)	28.65 ± 1.17a,d)	19.48 ± 1.3b,c)
20	72.04 ± 4.69f,g)	64.37 ± 4.60b,c)	45.45 ± 1.75a,d)	47.95 ± 4.22b,e)	35.93 ± 2.41b,c)
30	88.17 ± 5.38h,i)	75.86 ± 7.96g,j)	51.52 ± 8.75b,e)	54.39 ± 2.68b,e)	46.75 ± 3.44c,f)
40	91.40 ± 5.38i,k)	90.80 ± 5.75i,k)	73.74 ± 9.64f,g)	58.48 ± 9.99b,c)	56.71 ± 2.84g,j)
50	98.23 ± 1.07l)	93.10 ± 3.98k,l)	81.82 ± 7.63j,m)	68.42 ± 9.98c,f)	65.37 ± 3.46h,m)
60	86.02 ± 5.69h,i)	89.66 ± 7.96i,k)	90.91 ± 12.25i,k)	65.50 ± 4.57b,c)	64.93 ± 3.44h,i)

^a–m)^ Mean values with different superscripts are significantly (*p* ≤ 0.05) different from each other; observed values are expressed as mean ± standard error.

**Table 2:** Percentage removal of Lanasyn brown dye solution in different times by chemically modified sugarcane bagasse in column‐based filtration unit (temperature 30 ± 2 °C)

Time [min]	Concentration [ppm]				
	10	20	30	40	50
10	50 ± 2.41a)	50.62 ± 2.27b,c)	68.38 ± 5.20d,e)	63.64 ± 1.82d,e)	75.44 ± 7.09e,f)
20	54.17 ± 2.41a,d)	62.96 ± 5.66b,c)	86.32 ± 3.73b,c)	83.03 ± 9.17b,f)	95.91 ± 5.76c,g)
30	69.44 ± 1.39e,f)	85.18 ± 2.14c,g)	88.89 ± 3.73b,c)	90.91 ± 5.55b,c)	94.15 ± 6.43c,g)
40	87.5 ± 2.41b,c)	95.06 ± 6.53c,g)	94.87 ± 1.48g,h)	92.72 ± 1.82c,g)	95.91 ± 3.83c,g)
50	91.67 ± 4.17c,g)	97.53 ± 8.10c,g)	97.43 ± 0.0i,j)	93.33 ± 0.61g,h)	97.08 ± 1.55h,i)
60	90.28 ± 5.56b,c)	98.76 ± 1.23c,g)	95.73 ± 0.85k)	93.94 ± 3.03j,l)	97.66 ± 0.58k,l)

^a–l)^ Mean values with different superscripts are significantly (P ≤ 0.05) different from each other; observed values are expressed as mean ± standard error.

**Figure 2:**
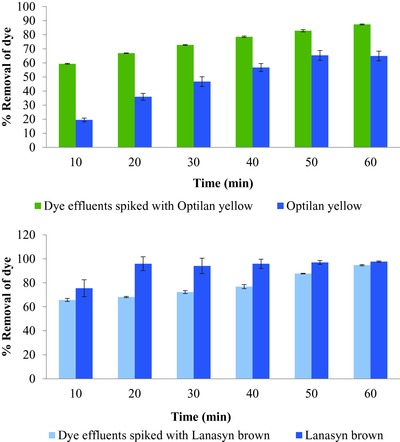
Percentage removal (±SE) of 50 ppm Optilan yellow and industry dye effluent spiked with 50 ppm Optilan yellow (above), and 50 ppm Lanasyn brown and industry dye effluent spiked with 50 ppm Lanasyn brown (below) in column‐based filtration unit (temperature 30 ± 2 °C).

Experiment using textile industry dye effluent spiked with 50 ppm Optilan yellow and only 50 ppm Optilan yellow showed similar trends of percentage removal of dye (>80% and > 60%, respectively) (**Figure** **2**, above). However, textile industry dye spiked with Optilan yellow showed higher removal as compared to only Optilan yellow. Whereas, for textile industry dye effluent spiked with 50 ppm Lanasyn brown and only 50 ppm Lanasyn brown showed similar trends of percentage removal of dye (>90% and >95%), respectively, in column‐based filtration unit (Figure 2, below). This suggests that the substances present in the dye effluents do not affect the performance of the column to a considerable extent.

### 2.2 Effect of Initial Dye Concentration

The initial dye concentration influences the removal efficiency of the bioadsorbent for dye removal.^27^, ^28^ Percentage removal of Optilan yellow and Lanasyn brown dye solutions (10–50 ppm initial concentration) at different times using sugarcane bagasse as an adsorbent in column‐based filtration unit is shown in Figure 1.

#### 2.2.1 Optilan Yellow

The removal efficiency exhibited a concentration‐dependent decrease in the range 10–5 ppm. Among the different concentrations of Optilan yellow, maximum removal of 98.23% was recorded at 10 ppm during 50 min time (Figure 1 and Table 1). The effect of the initial dye concentration depends on the relationship between the dye concentration and the available binding sites on an adsorbent surface.^29^, ^30^ At a lower concentration of dye, the availability of unoccupied binding sites on adsorbent is higher as compared to higher dye concentrations where most of the sites are occupied in a short duration of contact, and there is no significant increase in removal efficiency of the adsorbent after all the binding sites are engaged in binding with dye molecules.^31^

**Figure 3:**
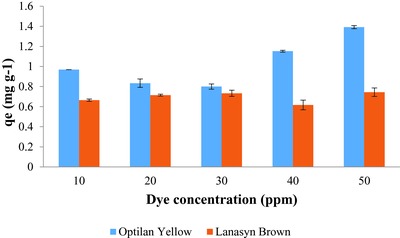
Equilibrium adsorption (*q*_e_) of chemically modified sugarcane bagasse for Optilan yellow and Lanasyn brown (conditions as for Figure 1).

The adsorption capacity (*q*_e_) for sugarcane bagasse varied in a concentration‐dependent manner for the initial concentration ranging from 10 to 50 ppm of Optilan yellow (**Figure** **3**). The equilibrium adsorption value (*q*_e_) increased in a concentration‐dependent manner except 20 and 30 ppm, where a decrease in adsorption capacity was recorded as compared to 10 ppm. Initially, *q*_e_ was less and gradually increased as treatment time increased to Optilan yellow. A steep increase in adsorption capacity was noticed at 50 ppm concentration as compared to a slight increase in other lower concentrations of dye concerning increasing time. This phenomenon can be explained by an earlier report of Bulut and Aydin^32^ who reported that the actual amount of dye adsorbed per unit mass of adsorbent increased with increase in dye concentration and this may be due to the high driving force for mass transfer at a high initial dye concentration.

#### 2.2.2 Lanasyn Brown

The initial concentration of dye also plays a significant role in the biosorption process. It acts as an important driving force to prevail over all mass transfer resistance of all dye molecules between aqueous and solid phases.^33^ The higher initial dye concentrations result in stimulation of biosorption process.^34^ This explanation is in agreement with our experimental results of metal complex dye, Lanasyn brown (Figure 1 and Table 2). It is evident from the data that as initial dye concentration increases, percentage removal of dye increases. Among the different concentrations of Lanasyn brown, maximum removal of 98.76% was recorded at 20 ppm during 60 min time. There was a significant reduction in the quantity of the dye throughout the experiment for all the five concentrations. Similar observations were reported by Deniz and Saygideger^35^ who worked on the biosorption of Basic Red 46 using princess tree leaf biomass and found an increase in biosorption of dye with the increase in initial dye concentration. Other researchers also reported the similar results for the removal of dyes from aqueous solutions.^36^, ^37^

The adsorption capacity (*q*_e_) for sugarcane bagasse varied in a concentration‐dependent manner for the initial concentration ranging from 10 to 50 ppm of Lanasyn brown (Figure 3). As concentration increased, there was zigzag pattern *q*_e_ recorded. Initially, *q*_e_ was less and gradually increased as time increased for Lanasyn brown dye. Similarly, Özacara and Sengil^38^ observed increasing *q*_e_ of pine sawdust when the initial concentration of metal complex blue and metal complex yellow dye increases.

### 2.3 Effect of Contact Time on Dye Removal

**Figure 4:**
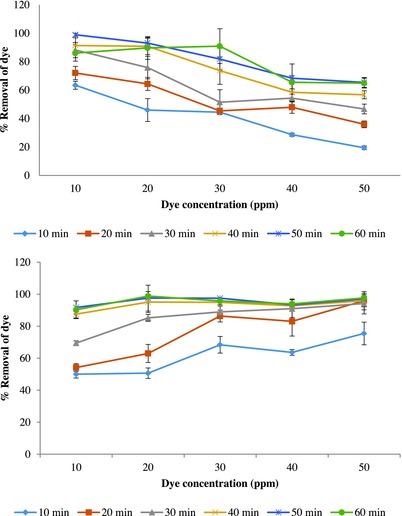
Effect of contact time on percentage removal (±SE) of Optilan yellow (above) and Lanasyn brown (below) by chemically modified sugarcane bagasse in column‐based filtration unit (temperature 30 ± 2 °C).

Effect of contact time with sugarcane bagasse on percentage removal of Optilan yellow dye solution (10–50 ppm initial concentration) at different times using sugarcane bagasse as an adsorbent in column‐based filtration unit is shown in (**Figure** **4**, above). Data showed that as time increases, percentage removal of dye increases for all the concentrations of dye solution. Percentage removal of dye recorded at initial 10 min range from 19% to 63% and after that, a gradual increase with time was noticed.

Percentage removal of Lanasyn brown dye solution at a different time is presented in Figure 4 (below). Data exhibited that initially, there was less removal of dye and gradual increase in percentage removal of dye with time. The rate of dye removal increases with contact time increasing to a certain extent. Further, it was also explained that due to the deposition of dyes on the available adsorption site on the adsorbent material, increase in contact time does not increase the uptake of dye.^39^

### 2.4 Changes in pH of Dye Solution

The pH is only the important factor in the adsorption process for dye. The pH of a solution/medium will manage the magnitude of electrostatic charges which are imparted by the ionized dye molecules. As a result, the rate of adsorption will vary with the pH of an aqueous medium.^40^ Changes in pH of dye solution during present adsorption study were determined by observing initial pH of the dye solution and final pH of effluent dye after completion of adsorption experiment for 1 h. Data show that there was an increase in pH after adsorption of dye in sugarcane bagasse (**Table** **3**). For Optilan yellow, initial pH was acidic at 10, 20 ppm and neutral to alkaline at 30, 40, and 50 ppm and became alkaline after the adsorption study. Whereas for Lanasyn brown, initial pH was acidic at 10, 20, and 30 ppm and alkaline at 40 and 50 ppm and became alkaline after the adsorption study. Generally, for low pH solution, the percentage removal of dye decreases for cationic dye adsorption, while for anionic dyes, the percentage removal of dye increases.^28^

**Table 3:** Changes in pH of dye solution during adsorption

Concentration of dye [ppm]	Optilan yellow		Lanasyn brown	
	Initial	Final	Initial	Final
10	6.5	7.6	6.7	7.9
20	6.2	7.3	6.3	7.5
30	7	7.5	6.8	7.9
40	7.7	8.2	7.6	8.1
50	7.2	8.6	7.4	8.2

### 2.5 Column Kinetic Study

The column adsorption data for 30 ppm Optilan yellow and 10 ppm Lanasyn brown dye concentration were selected and analyzed using Thomas, Yoon–Nelson, and Adams–Bohart models. And, textile industry dye effluent spiked with 50 ppm of each dye was also analyzed using these models.

#### 2.5.1 Thomas Model

The experimental data were fitted with Thomas model to investigate the breakthrough behavior of both the dyes and spiked textile industry dye effluent adsorption on the column (**Table** **4**). The linearized form of Thomas model was used to estimate the two unknown parameters *K*_th_ and *q*_max_.

**Table 4:** Thomas model parameters using linear regression analysis

Dyesa)	*C*_0_ [mg L^−1^]	*Q* [mL min^−1^]	*m* [g]	*K*_th_ [mL mg^−1^ min^−1^]	*q*_max_ (exp) [mg g^−1^]	*q*_max_ (cal) [mg g^−1^]	*R* ^2^
Optilan yellow	33	250	400	0.002	0.187	0.581	0.995
Lanasyn brown	24	250	400	0.002	0.137	0.176	0.904
Optilan yellow‐spiked textile industry dye effluent	681	250	400	0.00004	3.691	1.493	0.995
Lanasyn brown‐spiked textile industry dye effluent	478	250	400	0.00009	2.831	0.699	0.863

^a)^ *C*_0_ – initial dye concentration in mg L^−1^; *Q* – flow rate in mL min^−1^; *m* – adsorbent weight in g, *K*_th_ – Thomas model constant in mL mg^−1^ min^−1^; *q*_max_ (exp) – experimental maximum adsorption capacity in mg g^−1^; *q*_max_ (cal) – calculated maximum adsorption capacity in mg g^−1^; *R* ^2^ – correlation coefficient.

The results indicate that the experimental adsorption capacities and the adsorption capacities determined using Thomas model are nearly same. The values of *R* ^2^ were quite high. So, the experimental data are in good agreement with the theoretical data which are evident from high correlation coefficients and show good fitting of Thomas model (Table 4).

#### 2.5.2 Yoon–Nelson Model

The breakthrough behavior of both the dyes and spiked textile industry dye effluent adsorption on the column was also investigated by applying Yoon–Nelson model. The time required for 50% breakthrough was determined using Yoon–Nelson model by linear regression analysis. The unknown parameters of Yoon–Nelson model are presented in **Table** **5**.

**Table 5:** Yoon–Nelson model parameters using linear regression analysis

Dyesa)	*C*_0_ [mg L^−1^]	*Q* [mL min^−1^]	*K*_yn_ [min^−1^]	*q*_max_ (exp) [mg g^−1^]	*q*_max_ (cal) [mg g^−1^]	*R* ^2^
Optilan yellow	33	250	0.0724	0.187	0.581	0.996
Lanasyn brown	24	250	0.0542	0.137	0.176	0.905
Optilan yellow‐spiked textile industry dye effluent	681	250	0.0305	3.691	1.498	0.995
Lanasyn brown‐spiked textile industry dye effluent	478	250	0.0431	2.831	0.697	0.863

^a)^ *C*_0_ – initial dye concentration in mg L^−1^; *Q* – flow rate in mL min^−1^; *K*_yn_ – Yoon–Nelson rate constant in min^−1^; *q*_max_ (exp) – experimental maximum adsorption capacity in mg g^−1^; *q*_max_ (cal) – calculated maximum adsorption capacity in mg g^−1^; and *R* ^2^ – correlation coefficient.

The result shows that high value of correlation coefficients and nearly similar experimental and calculated adsorption capacities indicate that Yoon–Nelson model is the best fitted for explaining the kinetic behavior of the process.

#### 2.5.3 Adams–Bohart Model

The values of saturation dye concentration, *N*_0_, and Adams–Bohart kinetic constant, *K*_AB_ were determined by linear regression analysis. For Optilan yellow, the value of *R* ^2^ shows good linearity and good agreement of experiment data with predicted data with high correlation coefficients, whereas for Lanasyn brown and textile industry dye effluent, this model is the least fitted as the experimental and calculated values are quite different (**Table** **6**).

**Table 6:** Adams–Bohart model parameters using linear regression analysis

Dyesa)	*C*_0_ [mg L^−1^]	*U*_0_ [cm min^−1^]	*m* [g]	*K*_AB_ [mL mg^−1^ min^−1^]	*N*_0_ [mg L^−1^]	*R* ^2^
Optilan yellow	33	1.17	400	−0.001	7.404	0.966
Lanasyn brown	24	1.17	400	−0.002	−2.608	0.896
Optilan yellow‐spiked textile industry dye effluent	681	1.17	400	−0.00003	−499.5	0.994
Lanasyn brown‐spiked textile industry dye effluent	478	1.17	400	−0.00007	−147.3	0.833

^a)^ *C*_0_ – influent dye concentration in mg L^−1^; *U*_0_ – linear velocity of influent dye solution in cm min^−1^; *m* – weight of the adsorbent in g; *K*_AB_ – kinetic constant in mL mg^−1^ min^−1^; *N*_0_ – saturation concentration in mg L^−1^; and *R* ^2^ – correlation coefficient.

The best‐fitted model among these three models for both the dyes and textile industry dye effluent are Thomas and Yoon–Nelson models.

### 2.6 Breakthrough Pattern

**Figure 5:**
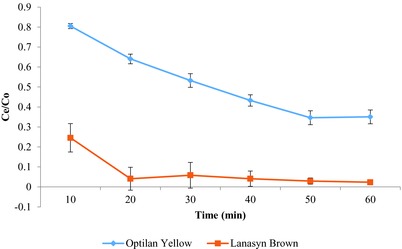
Breakthrough pattern (*C*_e_/*C*_0_) of Optilan yellow and Lanasyn brown during the treatment time of 1 h.

The breakthrough point (*C*_e_/*C*_0_ = 1.0) was reached for both the dyes after 60 min exposure time (**Figure** **5**). The dye Optilan red showed a value of 0.80 for *C*_e_/*C*_0_ after 10 min. However, there was a continuous decline in the value up to 50 min treatment time. The values for 50 and 60 min showed no considerable difference at 50 and 60 min. It is evident that minimum 10 min contact time with column material is required for binding of dye molecules with the column material. This indicates that the unoccupied binding sites on chemically treated bagasse require a certain degree of hydrous conditions for an effective binding with the dye molecules. The dye Lanasyn brown also exhibited a similar pattern with a little exception as the decline in the value of *C*_e_/*C*_0_ was as steep as noticed in Optilan yellow. The overall observations suggest that the column saturation for Lanasyn brown after 1 h treatment cycle was negligible (less than 10%) which indicates a considerably longer duration for reaching the breakthrough point. In contrast to Lanasyn brown, the column showed 34% and 35% (*C*_e_/*C*_0_ = 0.34–0.35) saturation after 50 and 60 min time, respectively. The comparative evaluation of both the dyes reveals that treated bagasse is more effective for the removal of Lanasyn brown from the aqueous medium.

### 2.7 Fourier Transform Infrared Spectroscopy (FTIR)

#### 2.7.1 Optilan Yellow

**Figure 6:**
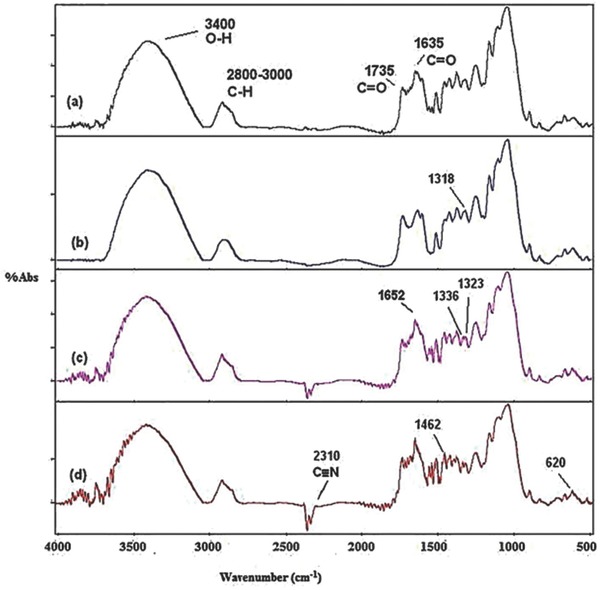
Stacked FTIR spectrum of a) SB, b) SB + acid pretreated, c) SB + Optilan yellow, and d) SB + acid pretreated + Optilan yellow (SB = sugarcane bagasse).

**Figure 7:**
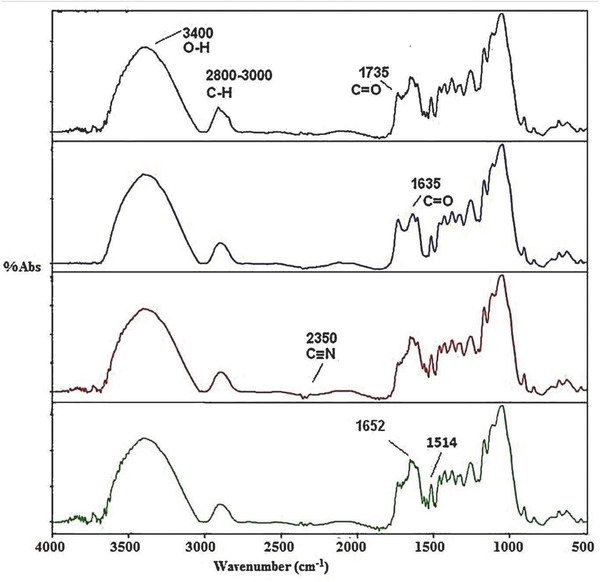
Stacked FTIR spectrum of a) SB, b) SB + acid pretreated, c) SB + Lanasyn brown, and d) SB + acid pretreated + Lanasyn brown textile dye.

**Figures** **6** and **7** show the stacked FTIR spectra of untreated and treated sugarcane bagasse with the textile dyes. The region between 3800 and 3000 cm^−1^ shows the related crystalline structure of cellulose. This region shows the sum of the vibration of valence bands of the hydrogen bond of the OH group and the bands of intramolecular and intermolecular hydrogen bonds.^41^, ^42^ The characteristic vibrational mode of C—H stretching of the hydroxyl group is seen around 2800–3000 cm^−1^ |in the FTIR spectra. The peak at 2850 cm^−1^ is due to the symmetric stretching of CH and CH_2_, while the peak at 2918 cm^−1^ is due to asymmetrical stretching of CH_2_ and CH. Both denote the characteristics of cellulose. The change in the pattern of vibrational changes after alginic acid treatment indicates lignin degradation, resulting in delignification of the bagasse.

#### 2.7.2 Lanasyn Brown

Figure 6 shows the stacked FTIR spectra of normal, alginic acid‐treated, Lanasyn brown‐treated chemically modified bagasse. It is evident from FTIR spectra that the acid treatment decreased the cellulose content which is evident from the increase in the intensity of vibrational changes in alginic treated samples as compared to control (untreated bagasse). After treatment of the combination of bagasse and alginic acid with Lanasyn brown dye, there was an increase in O—H and C—H groups' vibrations (Figure 7) which is clear indication of interaction between dye molecules with these functional groups. An increase in the intensity of peak around 1735 and 1635 cm^−1^ was noticed for the samples of chemically modified bagasse treated with Lanasyn brown dye. It is obvious from the spectra that there is a vibration of valence bands of the hydrogen bond of the OH group and the bands of intramolecular and intermolecular hydrogen bonds which results in the interaction of treated bagasse with Lanasyn brown.

Sugarcane bagasse as an agro‐waste material has little or no economic value, and it often creates a disposal problem. Therefore, utilization of this material has great significance for achieving a dual benefit objective which involves the safe utilization of agro‐waste in the process of contaminant remediation. Overall, this investigation will facilitate the judicious application of agro‐waste in general and sugarcane bagasse in particular, for the removal of the contaminants from water. Further, the approach presented here will facilitate the development of low‐cost technologies for remediation of environmental contaminants by utilizing the agro‐waste as adsorbents in a column bed.

## 3 Conclusion

Chemically modified sugarcane bagasse shows improved efficiency for textile dye removal when compared to untreated bagasse. The treated bagasse has a potentiality to replace the conventional adsorbents and therefore, in achieving a dual benefit by utilizing the agro‐waste for a useful purpose of textile dye remediation.

## 4 Experimental Section

*Preparation of Dye Solution*: Textile dyes with trade names Optilan yellow and Lanasyn brown were obtained from Archroma (Mumbai, India) for preparation of dye solution. Different concentrations of dye solution (5, 10, 15, 20, 25, 30, 35, 40, 45, 50 ppm) were prepared by dissolving the required quantity of dye powder in deionized water.

*Preparation of Adsorbents and Designing of Column Filtration Unit*: Sugarcane bagasse was collected from local sugarcane juice vendors and washed thoroughly in running water for 2–3 h to remove dirt, dust, and sugar traces. The washed bagasse was treated with KMnO_4_ (0.1%) solution for lignin oxidation and dried under the sun. The second step of drying was carried out in the oven at 120 °C till it dried completely. Once drying was completed, the bagasse was treated with 1% alginic acid (10 g L^−1^) for physical entrapment and dried in the sun and oven following the above procedure. This treated and dried bagasse was used for packing in the column filtration unit.

**Figure 8:**
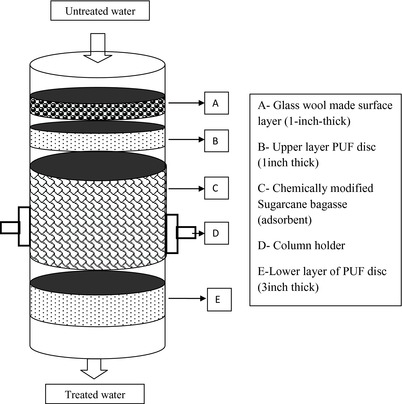
Expanded view of fixed‐bed column (PUF ‐ polyurethane foam).

Low cost and locally available materials like polyurethane foam (PUF), polyvinyl chloride (PVC) pipes, and plastic containers were used for the fabrication of the unit. The unit consisted of three components, viz., upper compartment, column bed, and lower compartment. Upper compartment served as the reservoir (25 L capacity) for the dye solution to be treated. The column was made of 6.5 in. diameter PVC cylinder pipe packed with a layer of sugarcane bagasse pretreated with alginic acid. The sugarcane bagasse layer was held between two layers of polyurethane foam (upper 1 in. and lower 3 in. thick, respectively, 200–300 µm pore size) to prevent the passage of particulate matter to treated water. The column was placed in a vertical orientation at the junction of upper and lower compartments of the device with the help of a column holder. A square‐shaped opening was made in the lower compartment at a distance 6–12 in. from the bottom which served as a window for the collection of samples at periodic intervals. Schematic design and exploded view of the column is presented in **Figure** **8**.

*Experiment*: For the experiment, 20 L of dye solution was filled in the upper compartment, and untreated water was passed for 1 h through the column with an average flow rate of 250 mL min^−1^. Samples were collected every 10 min interval in cleaned plastic bottles. Five different concentrations of dye, viz., 10, 20, 30, 40, and 50 ppm were used for the experiments. The dye solution was passed through the column for 60 min, and effluent dye solution was collected at a regular interval (10, 20, 30, 40, 50, and 60 min), and optical density (OD) of each dye at its respective absorption maxima (195 and 226 nm) was recorded. The concentration of dye was obtained using standard curve (10–100 ppm) curve. Initial pH and final pH (after 1 h experiment) were measured. All the experiments were carried out at room temperature (30 ± 2 °C). The removal efficiency^43^ of sugarcane bagasse at various time intervals for different dye concentrations in the column bed was calculated using the following equation

(1) $$\mathrm{Percentage}\;\mathrm{removal}\;R\;\left(\%\right)=\frac{(C_{i}-C_{c})\times \mathrm{100}}{C_{i}}$$

where *C*_i_ and *C*_c_ were the concentrations of dye solution before and after the treatment.

Equilibrium adsorption (*q*_e_) of sugarcane bagasse for the dye was calculated by using the equation

(2) $$q_{e}=\frac{\left(C_{0}\;-\;C_{i}\right)\times V_{i}}{W}$$

where *C*_0_ is the initial concentration of dye solution, *C_i_* is the concentration after “*i*th” time treatment, *V_i_* is the volume of the dye solution at *i*th time in a liter, and *W* is the weight of adsorbent in g.

*Modeling of Column Bed Adsorption*: Thomas model, Yoon–Nelson model, and Adams–Bohart models were used for evaluation of kinetic properties of the developed column.

*Modeling of Column Bed Adsorption—Thomas Model*: Thomas model^44^ assumes that dye migrates from the solution and diffuses through the surface of the adsorbent which leads to intraparticle diffusion and adsorption on active site. The linear form of Thomas model is presented by the equation

(3) $$\ln\;\left(\frac{C_{0}}{C_{t}}\;-\;1\right)\;=\;\frac{K_{\mathrm{th}}\;q_{\max}m}{Q}\;-\;K_{\mathrm{th}}C_{0}t$$

where *C*_0_ is the initial dye concentration in mg L^−1^, *C_t_* is the effluent concentration in mg L^−1^ at time *t*, *q*_max_ is the maximum adsorption capacity in mg g^−1^, *Q* is the flow rate in mL min^−1^, *m* is the adsorbent weight in g, *t* is the time in min, and *K*_th_ is the Thomas model constant in mL mg^−1^ min^−1^.

*Modeling of Column Bed Adsorption—Yoon–Nelson Model*: Yoon–Nelson model^45^ is used for predicting the time of column run before regeneration or replacement of column becomes necessary. It assumes that the rate of decrease in the probability of adsorption for each adsorbate molecule is proportional to the probability of adsorbate adsorption and the probability of adsorbate breakthrough on the adsorbent. The linear form of Yoon–Nelson model is given below

(4) $$\ln\;\left(\frac{C_{t}}{C_{0\;}-\;C_{t}}\right)\;=\;K_{\mathrm{yn}}t\;-\;K_{\mathrm{yn}}\tau$$

where *C*_0_ is the initial dye concentration in mg L^−1^, *C_t_* is the effluent concentration in mg L^−1^ at the time *t*, *t* is the time in min, τ is the time required for the 50% adsorbate breakthrough in min, and *K*_yn_ is the Yoon–Nelson rate constant in min^−1^.

The two unknown parameters of Yoon–Nelson equation, i.e., *K*_yn_ and τ were determined from the slope and intercept of the plot of $\ln\;\left(\frac{C_{t}}{C_{0}}\;-\;C_{t}\right)$ versus *t*. Based on Yoon–Nelson model, the amount of dye being adsorbed in a column is half of the total dye entering the column within 2τ period. For a given column

(5) $$q_{\max}\;=\;\frac{\frac{1}{2}C_{0}\frac{Q}{1000}2\tau }{m}$$

where *C*_0_ is the initial dye concentration in mg L^−1^, *Q* is the flow rate in mL min^−1^, and *m* is the total weight of adsorbent in g.

*Modeling of Column Bed Adsorption—Adams–Bohart Model*: Adams–Bohart model^46^ is based on the surface reaction theory which assumes that equilibrium is not instantaneous. Therefore, the rate of the adsorption is proportional to the adsorption capacity which remains on the sorbent. This approach focused on the estimation of characteristic parameters such as saturation concentration (*N*_0_) and kinetic constant (*K*_AB_). The linear form of Adams–Bohart model is given by the following equation

(6) $$\ln\;\left(\frac{C_{t}}{C_{0}}\right)\;=\;K_{\mathrm{AB}}\;C_{0}t\;-\;\frac{K_{\mathrm{AB}}N_{0}Z}{U_{0}}$$

where *C*_0_ and *C_t_* are the influent and effluent dye concentration in mg L^−1^, *K*_AB_ is the kinetic constant in mL mg^−1^ min^−1^, *U*_0_ is the linear velocity of the influent dye solutioncm min^−1^, *Z* is the bed depth of column in cm, and *N*_0_ is the saturation concentration in mg L^−1^. The values of *N*_0_ and *K*_AB_ were obtained from the intercept and slope of the linear plot of $\ln\;\left(\frac{C_{t}}{C_{0}}\right)$ against time *t*.

*FTIR Analysis*: FTIR characterization was carried out for the study on change of vibration pattern of various functional grow ups after treatment with the dyes. FTIR spectra of samples were recorded in the range from 400 to 4000 cm^−1^ with a scan rate of 4 cm^−1^ by FTIR spectrometer (Shimadzu 8400s, Japan). 5% of the bagasse (treated and untreated) and potassium bromide (KBr) powder (95%) were mixed mechanically into an agate mortar and ground to fine powder. This mixture was pressed into a pellet in the form of disk shape and scanned in the above range.

*Experiment with Textile Industry Dye Effluent*: Textile industry dye effluent was collected from a local dye house (Executive Garment processors Pvt. Ltd., MIDC, Mumbai) and spiked with 50 ppm Optilan yellow and 50 ppm Lanasyn brown dyes separately. The spiked dye effluent was passed through the column for 60 min, and effluent dye solution was collected at a regular interval (10, 20, 30, 40, 50, and 60 min), and OD of each dye at its respective absorption maxima (195 and 226 nm, respectively) was recorded. The experiments where dye‐house effluent was used for removal study showed a considerable decrease in total dissolved solids (TDS) before (281 ppm) and after filtration (84 ppm). However, the turbidity showed a negligible difference after the treatment of dye‐house effluent (92 NTU and 93 NTU; before and after filtration, respectively). It showed that the alginic acid‐treated bagasse remained sTable during the downflow of effluent water, indicating the negligible extent of leaching of alginic acid during the filtration process.

## Conflict of Interest

The authors declare no conflict of interest.
